# Inactivation Switch for Run-up in TRPV2

**DOI:** 10.21203/rs.3.rs-9726674/v2

**Published:** 2026-08-03

**Authors:** Guangyu Wang

**Affiliations:** 1 Department of Physiology and Membrane Biology, University of California School of Medicine, Davis, CA, USA; 2 Department of Drug Research and Development, Institute of Biophysical Medico-chemistry, Reno, NV, USA

**Keywords:** Allosteric gating coupling, dynamic interdomain interaction, digital biology, systematic thermal instability, temperature threshold, thermoring energetic structure, thermosensitivity

## Abstract

The homotetrameric thermosensitive transient receptor potential vanilloid 2 (TRPV2) channel exhibits both inactivation and run-up in response to heat or 2-aminoethoxydiphenyl borate (2-APB). Although a three-state model has been proposed to explain the run-up phenomenon, the role of inactivation remains unclear. In this computational study, a highly sensitive thermoring energetic model was employed to analyze and compare three-dimensional cryo-EM structures of TRPV2 in activated and inactivated states induced by different chemical perturbations. The analysis revealed that, in response to 2-APB or a mild detergent, the weakest tertiary bridge between the pre-S1 domain and the ankyrin repeat domain, which is present in the pre-open closed state, remains intact in the activated state but is disrupted in the inactivated state. In contrast, the highly conserved swapping π bridges near the lower gate are broken in the activated state but re-established in the inactivated state. Furthermore, the greater dynamic systematic thermal instability of the pre-open inactivated state compared with the activated state may account for similar, or mirrored, run-up responses induced by either 2-APB or heat. A stable pre-open activated state was also identified, exhibiting a lower activation threshold and reduced thermosensitivity that were well matched to facilitate subsequent heat activation and thereby enhance heat-induced run-up. These findings demonstrate that thermoring energetic analysis of protein structures in distinct functional states can precisely identify the dynamic allosteric communication networks that regulate protein activity.

## Graphical Abstract

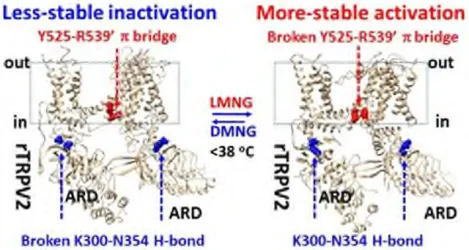

## INTRODUCTION

The regulation of multidomain, thermosensitive transient receptor potential (TRP) vanilloid 1–4 (TRPV1–4) channels by various physical and chemical stimuli is essential for maintaining organismal homeostasis and contributes to numerous physiological or pathological processes. These polytopic membrane proteins exhibit complex topology and assemble as homotetramers, with each subunit comprising an N-terminal ankyrin repeat domain (ARD), six transmembrane helices (S1–S6) and a C-terminal domain (CTD). The S1–S4 helices form the peripheral voltage sensor-like domain (VSLD), whereas the S5–S6 helices, together with the two pore turrets and the intervening pore helix (PH), constitute the central pore domain (PD). In addition, the pre-S1 domain linking the VSLD and the ARD, the S4–S5 linker connecting the VSLD and PD, and the conserved TRP domain located between the PD and the CTD form key interfacial elements that play pivotal roles in channel gating. Furthermore, the PD of each subunit undergoes domain swapping with the PD’ and the VSLD’ of the adjacent one, creating extensive intersubunit interactions. In TRPV2 and TRPV3, the pre-S1 domain also establishes an interprotomer interface with the neighboring ARD’. Together, these global interdomain and intersubunit allosteric networks coordinate channel gating by regulating two functional gates: the upper gate at the selectivity filter, defined by the conserved GXGY motif (X=M or L; Y=D or E) within the PH, and the lower gate formed by the LIA motif near the α-to-π helical transition of S6. These structural features collectively enable precise modulation of TRPV1–4 channel activity in response to a wide range of stimuli (1).

Notably, both mouse TRPV3 (mTRPV3) and rat TRPV2 (rTRPV2) channels exhibit use-dependent heat sensitization, reaching similar maximal activity during heat-induced run-up. In contrast, rat TRPV1 (rTRPV1) and TRPV4 (rTRPV4) undergo use-dependent heat desensitization and display distinct maximal activity during heat-induced run-down (2–12). An initial brief heat stimulus typically alters the temperature threshold and heat sensitivity of the subsequent one. Although the thermodynamic basis underlying the use-dependent heat responses of TRPV1 and TRPV3 has been well illustrated (13–17), the molecular mechanism responsible for heat-induced run-up in rTRPV2 remains much less well understood.

Given that rTRPV2 also exhibits a similar run-up in response to 2-aminoethoxydiphenyl borate (2-APB), an exogenous nonselective chemical agonist of TRPV1–3 channels (18), a three-state model (C_1_→C_2_↔O) has been proposed to explain the run-up induced by either heat or 2-APB. In this model, run-up is attributed to the lower thermodynamic stability of the second closed state (C2) relative to the open state (O), which favors channel opening. However, the distinct cross-use dependence of heat- and agonist-induced activation suggests that these stimuli engage different activation pathways. Specifically, prior heat stimulation markedly sensitizes subsequent agonist responses, whereas prior agonist stimulation does not similarly sensitize heat responses (6). Furthermore, activation of rTRPV2 by either 2-APB or heat is accompanied by channel inactivation (19, 20). Therefore, a unified gating model that incorporates both activation and inactivation is required to account for the similar run-up induced by heat and 2-APB.

The TRPV1–4 channels are activated above characteristic temperature thresholds (T_th_) ranging from 25 °C to 55 °C, together with the release of an inhibitory lipid or ligand from the vanilloid site or the VSLD (2–5, 13–14, 21–25). Although disrupting the distant weakest tertiary noncovalent interactions at distinct structural interfaces is required to initiate channel activation above these temperature thresholds, breaking the highly conserved swapping π bridges at the S4–S5 linker/S5’ interface primes the final activation of TRPV1, TRPV3, and TRPV4 (26–28). Thus, similar to other allosteric proteins (29–33), the temporal sequence from the initial thermal trigger to the distant effector site suggests an allosteric gating mechanism that couples disruption of the weakest and swapping bridges. These include the weakest Y401-R499/E406-K504 bridge and the swapping Y565-R579’ bridge in rTRPV1/human TRPV1 (hTRPV1), the weakest E467-K545 bridge and the swapping Y575-K589’ bridge in oxidized human TRPV3 (hTRPV3), and the weakest P498/Y502-Y567 bridge and the swapping Y602-R616’ bridge in human TRPV4 (hTRPV4). In contrast, during the initial thermal activation of rTRPV2 above 48–55 °C, coupling between the least stable K300-N354, S486-Y497, or L555-Y590 bridge and the conserved swapping H521-R539’-Y525-N639’ bridges near the lower gate is not strictly maintained. This difference arises, at least in part, because the weakest K300–N354 bridge is stabilized rather than disrupted in the cold-activated state, which functionally mirrors the highly thermosensitive heat-activated state (34).

On the other hand, disrupting the initial broken Y575-K589’ bridge in oxidized TRPV3 abolishes the critical weakest D586-T680 H-bond within the PD, leading to channel inactivation, pore dilation, and a tetramer-to-pentamer transition (27, 35–36). Similarly, chemical perturbation of the swapping H521-R539’-Y525-N639’ bridges near the lower gate by 2-APB also inactivates the rTRPV2 channel (37). In this regard, it is hypothesized that the weakest K300-N354 bridge is allosterically coupled to the highly conserved swapping bridges near the lower gate and contributes to rTRPV2 inactivation. Furthermore, because the inactivated state of rTRPV1, which exhibits lower systematic thermal instability, is responsible for heat-induced run-down (17), it is also hypothesized that the greater dynamic systematic thermal instability of the inactivated state relative to the activated state drives the run-up of rTRPV2 induced by either 2-APB or heat.

In this computational study, a highly sensitive thermoring energetic model recently developed (15–17, 26–27, 34, 38–46), was used to test the hypotheses described above. Analysis of the three-dimensional cryogenic electron microscopy (cryo-EM) structures of rTRPV2 in both 2-APB-activated and inactivated states, with or without additional perturbation by cannabidiol (CBD) or probenecid (PBC) at various sites, revealed that the weakest K300-N354 bridge remained intact in the activated states but was disrupted in the inactivated states. Consistent with this finding, the non-conductive cryo-EM structure of apo TRPV2, obtained after removal of two phosphatidylethanolamine (PE) lipids from the VSLD and vanilloid site by the mild detergent decyl maltose neopentyl glycol (DMNG), was identified as representing the inactivated state.

Furthermore, because the inactivated state exhibited greater dynamic systematic thermal instability than the activated state, a detailed run-up mechanism emerged to explain the self-potentiation of rTRPV2 activity in response to either 2-APB or heat. In addition, because the second heat activation reaches the same open state as the first while exhibiting a lower activation threshold and reduced thermosensitivity (6), a pre-open activated state was also identified with thermodynamic parameters consistent with enhanced heat-induced run-up.

Finally, it is proposed that rTRPV2 gating is regulated by ligand-free allosteric communication between the weakest tertiary noncovalent bridge at the pre-S1/ARD interface and the quaternary swapping interaction at the S4–S5 linker/S5’ interface. This mechanism is accompanied by state-dependent rearrangement of the highly conserved swapping π bridges at the S4–S5 linker/S5’ interface near the lower gate, providing a structural basis for coupling local tertiary interactions to global channel gating.

## METHODS

### Cryo-EM structures used

Five full-length 3D cryo-EM structures of apo rTRPV2 channels lacking PE in the VSLD and the vanilloid site were primarily analyzed to investigate the structural basis of 2-APB-induced inactivation of rTRPV2. The structures represented the 2-APB-activated, 2-APB-inactivated, 2-APB/CBD-activated, 2-APB/CBD-inactivated and 2-APB/PBC-activated states (PDB IDs: 7N0N, model resolution = 4.15 Å; 7N0M, 3.5 Å; 7T37, 3.7 Å; 7T38, 3.8 Å; and 9B3V, 4.2 Å, respectively). These channels were purified in the DMNG detergent and subsequently reconstituted in membrane scaffold protein 2N2 (MSP2N2) nanodiscs at 4 °C (37, 47).

In addition, full-length or truncated 3D cryo-EM structures of other native TRPV2 channels were also analyzed to evaluate the proposed inactivation motif. The corresponding PDB IDs were 5AN8, 5HI9, 6BO4, 6U84, 6U86, 6U8A, 6U88, 6WKN, 7XEM, 7XEO, 7XEU, 7XEV, 7YEP, 7ZJD, 7ZJE, 7ZJG, 7ZJI, 7ZJH, 8EKP, 8EKQ, 8EKR, 8EKS, 8FFL, 8FFM, 8SLX, 8SLY and 9B3U (47–57).

### Detecting and filtering tertiary noncovalent interactions

Along the same polypeptide chain spanning residues from V254 to P726 of rTRPV2 (34), paired tertiary noncovalent bridges were sampled using UCSF Chimera according to the same strict and consistent criteria previously validated (15–17, 26–27, 34, 38–46). With the exception of transient fluctuation-induced perturbations, the filtering parameters used to identify these tertiary bridges were provided in the online Supporting Information (Tables S1–S4).

### Mapping tertiary thermoring energetic structures using the grid thermodynamic model

Although allostery has increasingly been exploited to regulate protein activity and stability and to facilitate drug discovery (29–33, 58–66), random thermal fluctuations between the low-energy native folded state and sparsely populated higher-energy unfolded or excited state can also perturb entire secondary structural elements whose intrinsic stability is lower than that of the overall fold of the isolated domain (67). Consequently, identifying a specific thermosensor that functions as an allosteric site to initiate cooperative heat- or cold-induced conformational responses remains a major challenge.

Because meridians—an energy network concept used in traditional medicine in China several thousands of years ago —were historically used as a means by which stimulation of a distant superficial acupuncture point could influence the function of an internal organ, a comparable and simple yet highly sensitive energy network is needed to describe allosteric regulation of protein activity through environmental temperature perturbations.

Similarly, just as frontier molecular orbital theory has been applied to explain pericyclic reactions in organic chemistry (68), the grid thermodynamic model, combined with Graph theory and the Floyd–Warshall algorithm (69), was applied to construct the systematic fluidic, grid-like tertiary noncovalent interaction mesh network termed a thermoring structure. This framework generates an energetic landscape that enables precise evaluation of the local stability of individual thermeorings, ranked from the biggest to the smallest. Through this analysis, the weakest tertiary bridge can be identified as a potential allosteric thermosensor that initiates global protein conformational changes when the temperature exceeds a specific matched melting temperature threshold (T_m,th_) (15–17, 26–27, 34, 38–46). Using the same workflow (34), this model was further applied to map the tertiary thermoring energetic architectures of rTRPV2 across different gating states.

**Figure 1.**
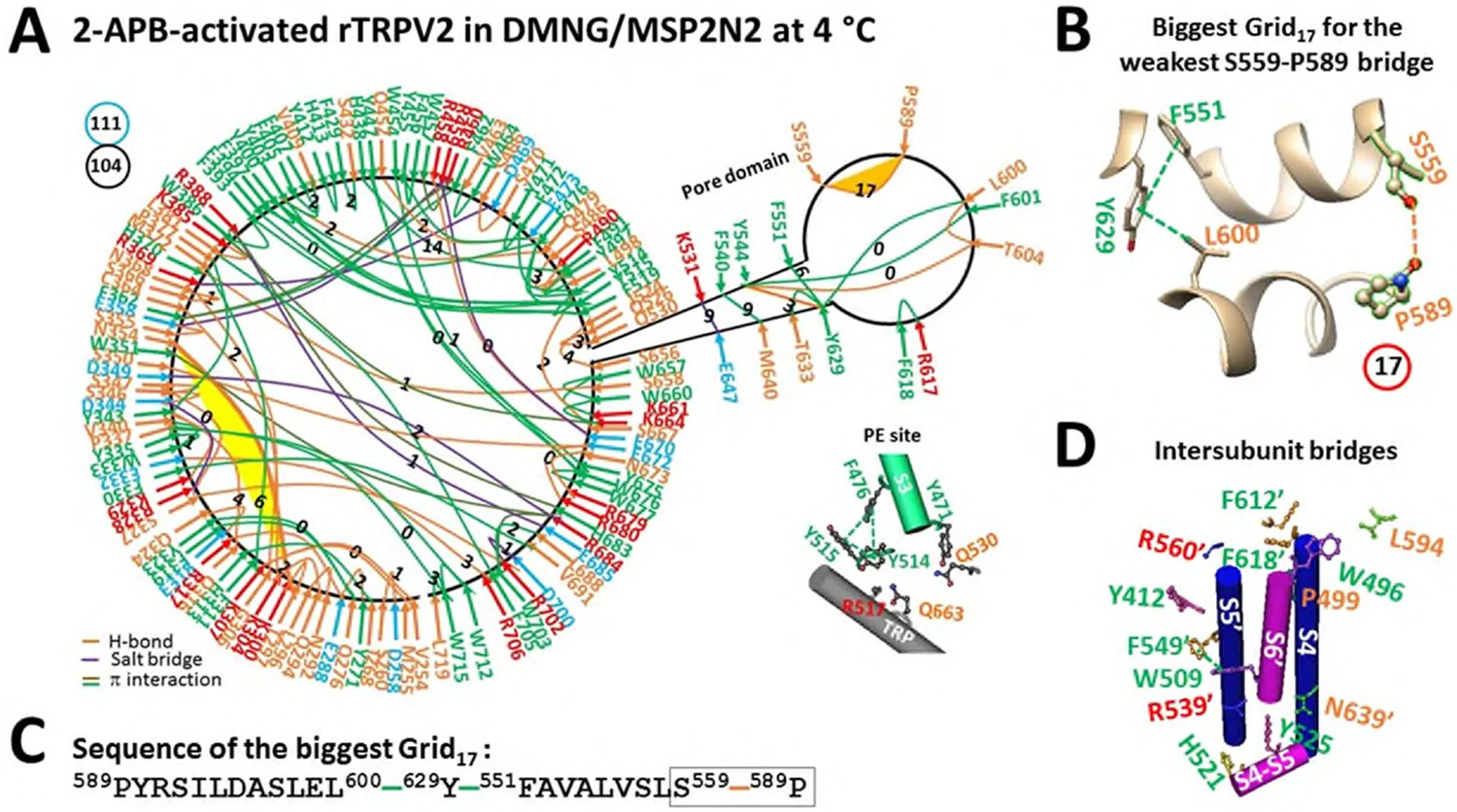
Thermoring structures of 2-APB-activated rTRPV2 at low temperature. (**A**) Grid-like mesh network of noncovalent interactions along the PE-dependent minimal gating pathway within a single subunit of 2-APB-activated rTRPV2 in a DMNG/MSP2N2 nanodisc at pH 8 and 4 °C (PDB: 7N0N). The same colored residues and arrows are used for convenient tracking. Salt bridges, π interactions, and H-bonds between paired amino acid side chains along the PE-dependent minimal gating pathway from V254 to P726 are shown as purple, green, and orange lines, respectively. The specific constrained grid sizes necessary to regulate the least-stable noncovalent interactions within each grid are indicated with black numbers. The identified least-stable S559-P589 H-bond within the biggest Grid_17_ is highlighted in orange. The previously identified weakest K300-N354 H-bond in the preopen closed state is highlighted in yellow. The total grid sizes and the total number of grid size-controlled noncovalent interactions along the PE-PEdependent minimal gating pathway are indicated in cyan and black circles, respectively. The inset shows the PE sites within the VSLD and the vanilloid site. (**B**) Structure of the biggest Grid17 with a 17-residue size to regulate the weakest S559-P589 H-bond at the external interface of two pore turrets. (**C**) Amino acid sequence of the biggest Grid17 to control the weakest S559-P589 bridge highlighted in the blue box. (**D**) Swapped interactions at the VSLD/PD’/PD interfaces near the PE site and upper and lower gates in the 2-APB-activated state.

As a result, after all involved noncovalent bridges were labeled according to their unique grid sizes or the minimal free residues contained within each grid, the weakest tertiary bridge—typically located in the primary or secondary biggest thermoring involving the domain-domain interface —could be identified as a potential allosteric site for initiating heat activation above the calculated T_m,th_. For example, the weakest S559-P589 H-bond at the interface between two pore turrets in 2-APB-activated rTRPV2 was identified as an allosteric thermosensor. Its T_m,th_ was determined by the biggest Grid_17_, which contained 17 free residues defining the grid size. These residues included the segments ^590^YRSILDASLE^599^ and the ^552^AVALVSL^558^ (Figure 1A). In parallel, the total numbers of noncovalent interactions (*N*) and the total grid sizes (*S*), along the same polypeptide chain were obtained for subsequent calculations.

### Calculating the dynamic systematic thermal instability

The dynamic systematic thermal instability (T_i_) was determined based on the following expression, as previously validated and tested in earlier studies (15–17, 26–27, 34, 38–46):

(1) $${\text{T}}_{\text{i}}=S/N$$

### Calculating the melting temperature threshold for heat unfolding

The melting temperature threshold (T_m,th_) for thermal unfolding of the least-stable noncovalent interaction within a specific grid at a physiological salt concentration of 150 mM NaCl was estimated using the following equation as previously described (15–17, 26–27, 34, 38–46):

(2) $${\text{T}}_{\text{m,th}}({}^{\circ}\text{C})=34+\left(\text{n}-2\right)\times 10+\left(20-\text{s}\right)\times 2$$

where, n represents the total number of basic H-bonds (approximately 1 kcal/mol each in a hydrophilic environment) that are energetically equivalent to the weakest noncovalent bridge governed by the corresponding grid, and s represents the size of the grid controlling the weakest tertiary bridge. According to this relationship, the static thermal stability of a grid increases with either a smaller grid size or a greater number of energetically equivalent basic H-bonds.

**Figure 2.**
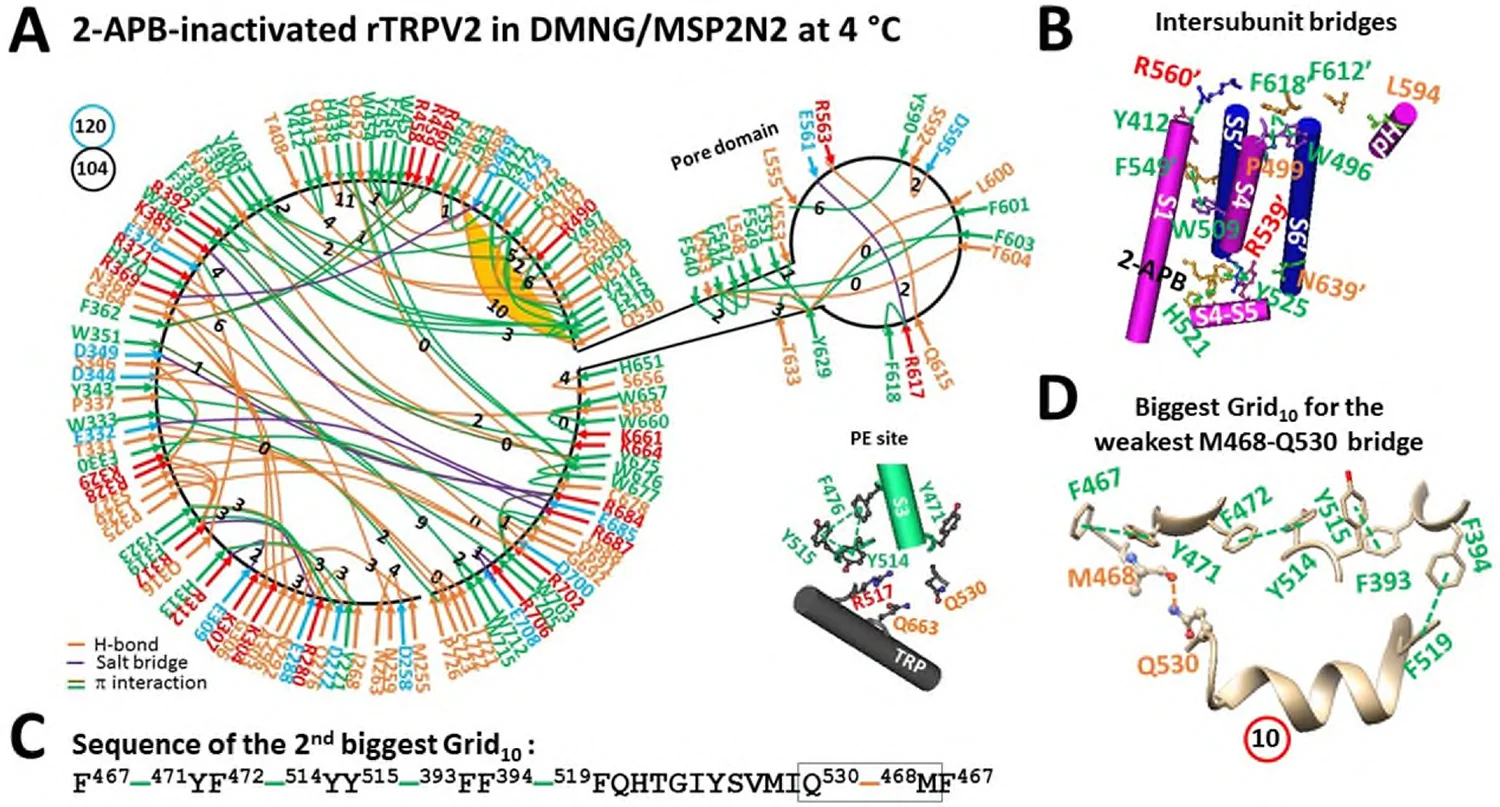
Thermoring structures of 2-APB-inactivated rTRPV2 at a low temperature. (**A)** Grid-like mesh network of noncovalent interactions along the PE-dependent minimal gating pathway within a single subunit of 2-APB-inactivated rTRPV2 in a DMNG/MSP2N2 nanodisc at pH 8 and 4 °C (PDB: 7N0M). The same colored residues and arrows are used for convenient tracking. Salt bridges, π interactions, and H-bonds between paired amino acid side chains along the PE-dependent minmal gating pathway from V254 to P726 are shown as purple, green, and orange lines, respectively. The specific constrained grid sizes necessary to regulate the least-stable noncovalent interactions within each grid are indicated with black numbers. The identified least-stable M468-Q530 H-bond within the 2nd biggest Grid_10_ is highlighted in orange. The total grid sizes and the total number of grid size-controlled noncovalent interactions along the PE-dependent minimal gating pathway are displayed in cyan and black circles, respectively. The inset shows the PE sites within the VSLD and the vanilloid site. (**B)** Swapped interactions at the VSLD/PD’/PD interfaces near the PE site and upper and lower gates in the 2-APB-inactivated state. (**C)** Structure of the 2nd biggest Grid_10_ with a 10-residue size to regulate the weakest M468-Q530 H-bond at the internal S3–S5 interface. (**D**) Amino acid sequence of the 2nd biggest Grid_10_ to control the weakest M468-Q530 bridge highlighted in the blue box.

For example, the Q414-H438-R490 π interactions in Figure 2A were stabilized by the first biggest Grid_11_ via a thermoring from Q414 to H413, Y412, T408, W509, G508, Y400, F476, Q479, S504, Y497, R490, H438, and back to Q414 (Figure 2A). Because the two π interactions within this thermoring were energetically equivalent to about 3 basic H-bonds (3 kcal/mol), the calulcated T_m,th_ required for disrupting either interaction was approximately 62 °C. In contrast, the predicted T_m,th_ for disrupting the M468-Q530 H-bond within the second biggest Grid_10_ was only 49 °C (Table 1). Therefore, among the tertiary noncovalent interactions along the polypeptide chain spanning residues from V254 to P726, the M468-Q530 H-bond represents the weakest tertiary bridge, rather than the Q414-H438 or H438-R490 interaction.

### Evaluating the systematic temperature sensitivity of rTRPV2

The grid-based structural thermo-sensitivity (Ω_10_) of a single ion channel during cold activation was defined and calculated according to previously established approaches (11–13, 21–24, 29).

(3) $$\Omega_{10}={\left[\left({\text{S}}_{\text{c}}-{\text{S}}_{\text{o}}\right)\text{E}/2\right]}^{\left(\text{Hc}/\text{Ho}\right)}={\left[\left({\text{S}}_{\text{c}}-{\text{S}}_{\text{o}}\right)\text{E}/2\right]}^{\left[\left(\text{ENc}/\text{ENo}\right)\right]}={\left[\left({\text{S}}_{\text{c}}-{\text{S}}_{\text{o}}\right)\text{E}/2\right]}^{\left(\text{Nc}/\text{No}\right)}$$

where, along the same polypeptide chain spanning residues from V254 to P726 in the closed and open states, N_c_ and N_o_ represent the total numbers of noncovalent interactions; H_c_ and H_o_ denote the total enthalpy contributions associated with these interactions; and S_c_ and S_o_ indicate the total grid sizes, respectively. The average energy intensity of a tertiary noncovalent interaction is represented by (E), which is generally assumed to be 1 kcal/mol (70). Thus, the mean Ω_10_ value reflects the thermally induced change in the chemical potential of structural grids resulting from alterations in the total enthalpy associated with noncovalent interactions during the transition from the closed to the open state along the same polypeptide chain. In this study, the non-conducting pre-open inactivated (7N0M or 5HI9) and activated (PDB ID: 9B3V) structures were considered as closed states for the corresponding calculations.

For comparison with structural thermo-sensitivity, the functional thermo-sensitivity (Q_10_) of a single ion channel for heat activation was calculated using the conventional relationship:

(4) $${\text{Q}}_{10}={\left({\text{X}}_{2}/{\text{X}}_{1}\right)}^{10/\left(\text{T}2-\text{T}1\right)}$$

where, X_1_ and X_2_ represent the measured functional responses at T1 and T2 (measured in Kelvin), respectively.

## RESULTS

### Identification of the unstable intermediate state for 2-APB-induced run-up

In the presence of 1 mM 2-APB, only activated and inactivated states were observed at 4 °C (37). Therefore, it is of particular interest to determine whether the inactivated state represents the unstable intermediate responsible for 2-APB-induced run-up. Along the PE-dependent minimal gating pathway from N254 to P726 in the 2-APB-activated state without PE in both the VSLD and vanilloid site (PDB ID: 7N0N), a total of 104 tertiary noncovalent interactions formed a grid-like mesh network consisting of 111constrained grid sizes (Figure 1A, Table S1). The resulting systematic thermal instability (T_i_) was thus calculated as 1.07 (Table 1).

Among these grids, the biggest Grid_17_ was identified as controlling the weakest S559-P589 H-bond through a thermoring pathway from P589 to L600, Y629, Y551, S559 and back to P589 (Figure 1B–C). Because this weakest bridge was energetically equivalent to 1.2 basic H-bonds (1.2 kcal/mol), the calculated melting temperature threshold (T_m,th_) was approximately 32 °C. Although this value falls within the temperature range reported for 2-APB-induced run-up (22–48 °C) (Table 1) (6), it defines 32 °C as the maximal temperature limitation for this process.

Notably, 2-APB-mediated perturbation of the swapping interface between the S4–S5 linker and S5’ disrupted the swapping H521-R539’-Y525-N639’ π bridges near the lower gate. This disruption was accompanied by the loss of Y412-R560’, F618’-W496/P499 and F612’-L594 π interactions near the upper gate (Figure 1D) (34).

In contrast, in the 2-APB-inactivated state without PE in both the VSLD and vanilloid site (PDB ID: 7N0M), the total number of tertiary noncovalent interactions remained unchanged; however, the summed grid sizes along the same PE-dependent minimal gating pathway from N254 to P726 increased from 111 to 120 (Figure 2A; Table S2). Consequently, the T_i_ increased from 1.07 to 1.15 (Table 1). Although the H521-R539’-Y525-N639’ π bridges associated with swapping near the lower gate remained disrupted by 2-APB binding, the Y412-R560’ and F618’-W496/P499 and F612’-L594 π interactions near the upper gate were restored relative to the pre-open closed state (Figure 2B) (34).

Meanwhile, the biggest Grid_10_ was found to regulate the least-stable M468-Q530 H-bond through a thermoring from F467 to Y471, F472, Y514, Y515, F393, F394, F519, Q530, M468, and back to F467 (Figure 2C–D). Because this weakest bridge at the S4/S5 interface was energetically equivalent to 1.5 basic H-bonds (1.5 kcal/mol), the calculated theoretical T_m,th_ was approximately 49 °C. This value remained within the temperature range (22–48 °C) associated with 2-APB-induced run-up and was consistent with the experimentally determined threshold for heat activation following saturated 2-APB treatment (Table 1) (6). Taken together, these findings suggest that the inactivated state, characterized by a higher dynamic T_i_ and a matched T_m,th_, may represent an unstable intermediate state underlying 2-APB-induced run-up (6).

Although heat activation of rTRPV2 is accompanied by inactivation (19–20), the channel still exhibits a similar run-up phenomenon (6). This raises the question of whether the heat-inactivated state represents an unstable intermediate responsible for heat-induced run-up. On the other hand, following perturbation with the mild detergent lauryl maltose neopentyl glycol (LMNG), the mirrored cold-evoked open state has been identified (34). Therefore, according to the heat capacity model (71), if a cold-inactivated state exists with a higher T_i_ than the cold-activated state, this difference should be detectable upon treatment with a similar mild detergent at low temperatures.

### Identification of a cold-inactivated state with a higher systematic thermal instability

A recent study demonstrated that PBC interacts with K118, L126, Y162, H165, I170 and K174 from one rTRPV2 monomer, as well as W333’ and Y335’ from the adjacent monomer, thereby preventing rTRPV2 inactivation (37, 47). Therefore, it is important to examine the domain-swapping interactions at the PBC site in both the activated and inactivated states in the presence of the agonist 2-APB, with or without CBD or PBC.

In the 2-APB-activated state (PDB ID: 7N0N), aside from the Y162-F161-F198-F199 π interactions, the inactivation-sensitive H165 also formed π interactions with the nearby H169 and F199 from the same subunit, as well as with the heat-sensitive Y335’ residue from the adjacent subunit (37, 72). By contrast, the 2-APB-inactivated state (PDB ID: 7N0M) contained additional Y335’-F198/F199 and W333’-Y162 π interactions, all of which were disrupted in the 2-APB/PBC-activated state. These observations suggest that a tighter domain-swapping interface at the PBC site seemed to favor the inactivated conformation.

**Figure 3.**
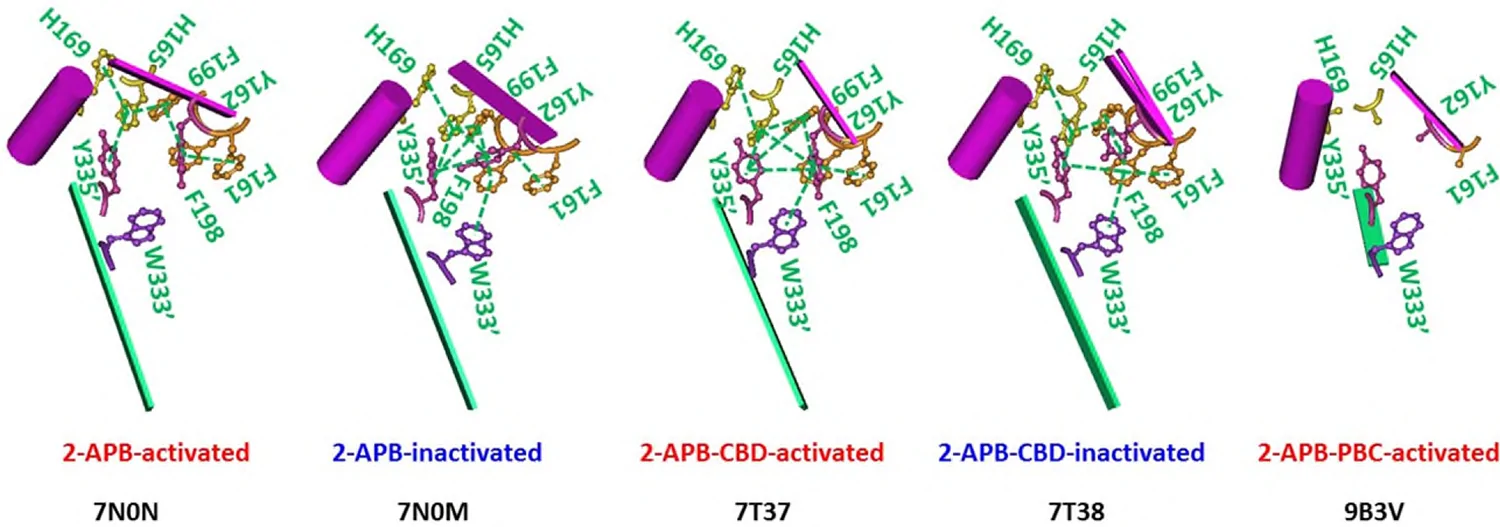
Comparison of intersubunit interactions in the activated and inactivated states of rTRPV2 with different agonists at low temperatures. The cryo-EM structures of rTRPV2 in response to various agonists at 4 °C (PDB ID: 7N0N, 7N0M, 7T37, 7T38 and 9B3V) were used for the model.

However, similar domain-swapping interactions were also observed in both the 2-APB/CBD-activated and inactivated states (Figure 3). Accordingly, the domain-swapping interface at the PBC-binding site alone cannot fully account for TRPV2 inactivation. It is necessary to define other structural determinants that underlie TRPV2 inactivation.

Given that interdomain interactions play a critical role in regulating channel gating, 29 domain-domain interactions were first identified in both the 2-APB-activated and inacted states. Furthermore, 12 and 13 unshared interactions were identified in the 2-APB-activated and inactivated states, respectively. Following additional CBD perturbation, only 4 and 6 shared interactions remained in the 2-APB/CBD-activated and inactivated states, respectively. Finally, the unique K300-N354 H-bond was also observed in the 2-APB/PBC-activated state (Table 2). Therefore, disrupting this K300-N354 weakest H-bond, which was identified in the pre-open closed state, may contribute to channel inactivation (34). This interpretation is consistent with the significant 4.4 Å downwards movement of the ARDs observed in the 2-APB-inactivated state (37).

Further structural analysis uncovered that the K300-N354 bridge was also present with other agonists, including protons, phytocannabinoid Δ9-tetrahydrocannabiorcol (C16) and estradiol (E2), either alone or in combination with PBC. Notably, in the absence of any agonist ligand, this bridge was still observed in three PE bound closed state structures (PDB ID: 8EKP, 8EKQ and 8EKR), one PE-free closed state structure (PDB ID: 6U86), and one PE-free open state structure (PDB ID: 6BO4) (Table 3) (50, 51, 57). In contrast, the bridge was absent in two PE-free apo rTRPV2 structures: one solubilized in DMNG (PDB ID: 5HI9) and the other subsequently reconstituted in MSP2N2 nanodiscs (PDB ID: 6U84) (Table 3) (49, 51). It was also absent in a truncated rabbit TRPV2 structure lacking PE in both the VSLD and vanilloid site (PDB: 5AN8) (Table 3) (48). Because DMNG is, like LMNG, a relatively mild detergent, it would be informative to determine whether the DMNG-inactivated state (PDB ID: 5HI9) exhibits greater systematic thermal instability than the LMNG-opened state (PDB ID: 6BO4) (PDB ID: 6BO4) (34).

**Figure 4.**
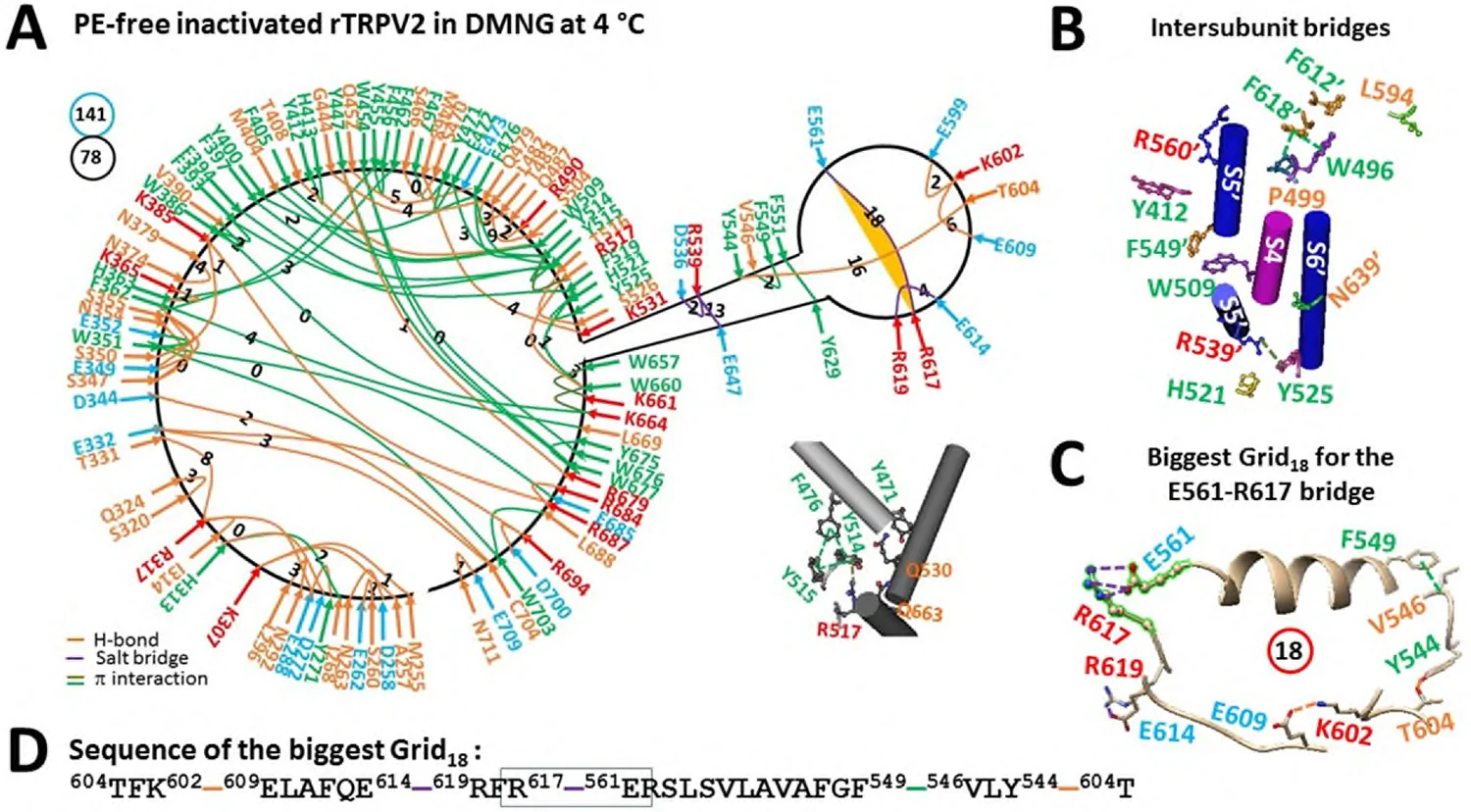
Thermoring structures of ligand-free inactivated rTRPV2 at a low temperature. (**A)** Grid-like mesh network of noncovalent interactions along the PE-dependent minimal gating pathway within a single subunit of ligand-free inactivated rTRPV2 in DMNG at pH 8 and 4 °C (PDB: 5HI9). The same colored residues and arrows are used for convenient tracking. Salt bridges, π interactions, and H-bonds between paired amino acid side chains along the PE-dependent gating pathway from V254 to P726 are shown as purple, green, and orange lines, respectively. The specific constrained grid sizes necessary to regulate the least-stable noncovalent interactions within each grid are indicated with black numbers. The identified least-stable E561-R617 salt bridge within the biggest Grid_18_ is highlighted in orange. The total grid sizes and the total number of grid size-controlled noncovalent interactions along the PE-dependent minimal gating pathway are displayed in cyan and black circles, respectively. The inset shows the PE sites within the VSLD and the vanilloid site. (**B)** Swapped interactions at the VSLD/PD’/PD interfaces near the PE site and upper and lower gates in the DMNG-inactivated state. (**C)** Structure of the biggest Grid_18_ with an 18-residue size to regulate the weakest E561-R617 salt bridge at the external interface of two pore turrets. (**D**) Amino acid sequence of the biggest Grid_18_ to control the weakest E561-R617 salt bridge highlighted in the blue box.

In the DMNG-inactivated state, a total of 78 tertiary noncovalent bridges and 141 grid sizes were identified along the same PE-dependent minimal gating pathway from V254 to P726 (Figure 4A, Table S3). Compared with the pre-open closed state, the W496-F618’-P499 bridges near the upper gate and the Y525-R539’ bridge near the lower gate were restored, whereas the swapped H521-R539’ and Y525-N639’ π bridges remained disconnected near the lower gate (Figure 4B) (34). In this case, relative to the LMNG-opened state (34), the grid-based systematic thermal instability (T_i_) increased from 1.71 to 1.81 (Table 1). In addition, the new weakest salt bridge, E561-R617, was identified at the external interface between two pore turrets (Figure 4A). This bridge was controlled by the biggest Grid_18_ via a thermoring from T604 to K602, E609, E614, R619, R617, E561, F549, V546, Y544, and back to T604 (Figure 4C–D). For the weakest E561-R617 bridge to be energetically equivalent to 2.0 basic H-bond (2.0 kcal/mol), the calculated T_m,th_ was 38 °C. This value is lower than the 41 °C predicted for the LMNG-opened state and well below the 48–55 °C range at which heat activation begins (Table 1) (20, 34). Accordingly, the DMNG-inactivated state, with its higher T_i_ value of 1.81, may represent a cold-inactivated intermediate that mirrors the heat-induced run-up process (6).

### Identification of an activated state that enhances heat-induced run-up

Although heat-induced run-up is enhanced by use-dependent activation, which exhibits a lower temperature threshold of 31–40 °C and a reduced thermosensitivity of 3.89 (6), the calculated structural thermosensitivity (Ω_10_) of 88.5 for the putative cold activation transition from this DMNG-inactivated state to the LMNG-opened state is still substantially higher than the experimentally determined Q_10_ of 3.89 (Table 1). Therefore, it is still necessary to identify an alternative pre-open activated state.

On the other hand, although the PBC-activated state has a total of grid sizes (~92) similar to that of the LMNG-opened state, resulting in a relatively low calculated thermosensitivity (Ω_10_= −1.84) (34), its predicted T_m,th_ of 46 °C is inconsistent with the experimentally observed threshold of 31–40 °C (6). In that regard, a structurally similar activated state induced by 2-APB/PBC was investigated as a potential pre-open activated state that could better account for the experimentally observed use-dependent enhancement of heat-induced run-up.

**Figure 5.**
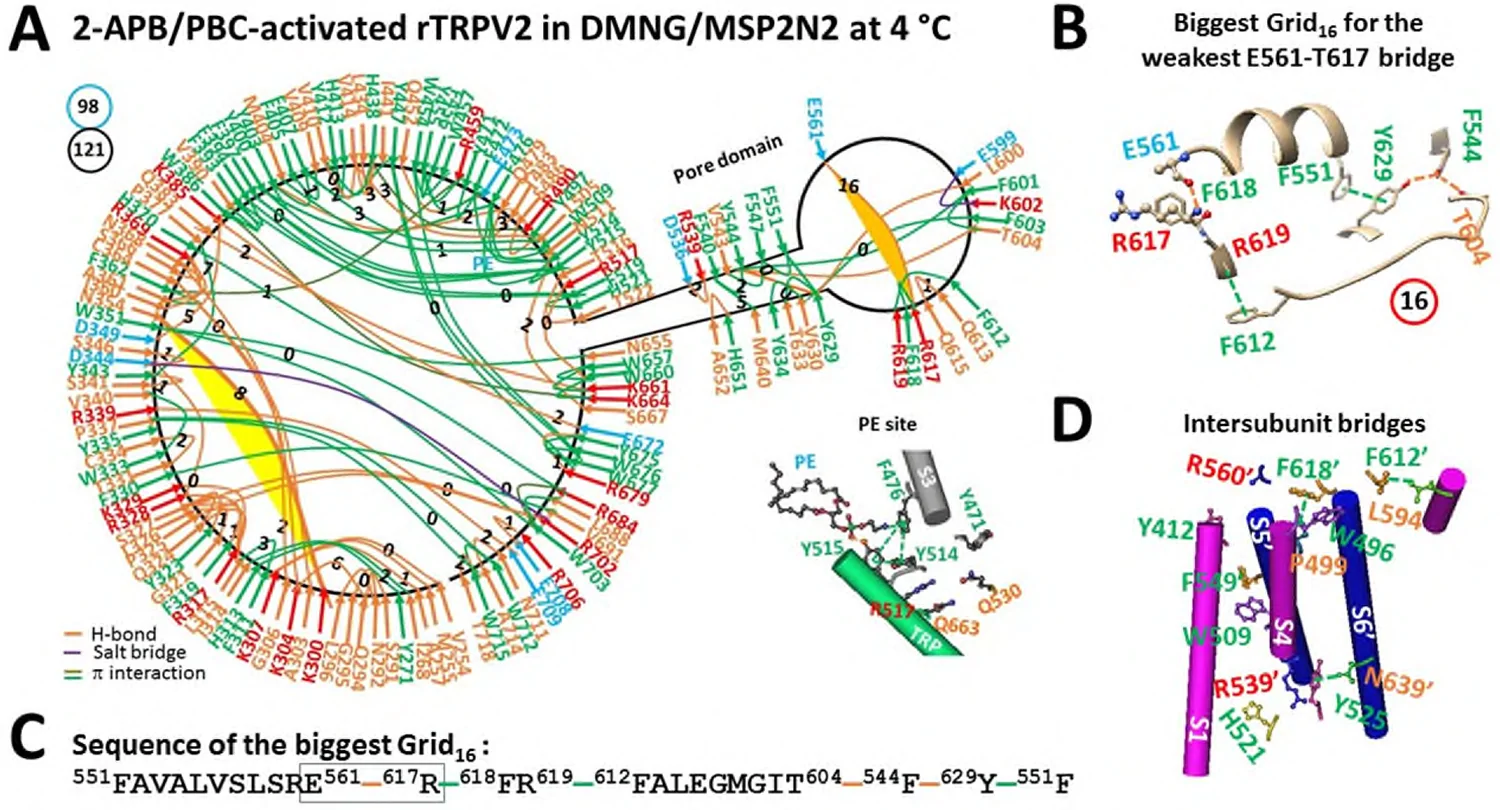
Thermoring structures of 2-APB/PBC-activated rTRPV2 at a low temperature. (**A)** Grid-like mesh network of noncovalent interactions along the PE-dependent minimal gating pathway within a single subunit of 2-APB/PBC-activated rTRPV2 in a DMNG/MSP2N2 nanodisc at pH 8 and 4 °C (PDB: 9B3V). The same colored residues and arrows are used for convenient tracking. Salt bridges, π interactions, and H-bonds between paired amino acid side chains along the PE-dependent gating pathway from V254 to P726 are shown as purple, green, and orange lines, respectively. The specific constrained grid sizes necessary to regulate the least-stable noncovalent interactions within each grid are indicated with black numbers. The identified least-stable E561-R617 H-bond within the biggest Grid_16_ is highlighted in orange. The previous weakest K300-N354 H-bond as revealed in the pre-open closed is also highlighted in yellow. The total grid sizes and the total number of grid size-controlled noncovalent interactions along the PE-dependent minimal gating pathway are displayed in cyan and black circles, respectively. The inset shows the PE sites within the VSLD and the vanilloid site. (**B)** Structure of the biggest Grid_16_ with a 16-residue size to regulate the weakest E561-R617 H-bond at the external interface of two pore turrets. (**C)** Amino acid sequence of the biggest Grid_16_ to control the weakest E561-R617 bridge highlighted in the blue box. (**D**) Swapped interactions at the VSLD/PD’/PD interfaces near the PE site and upper and lower gates in the 2-APB/PBC-activated state.

In this activated state, a total of 121 tertiary noncovalent interactions and 98 grid sizes were identified along the same PE-dependent minimal gating pathway from V254 to P726 (Figure 5A, Table S4). Correspondingly, the systematic thermal instability (T_i_) decreased significantly from 1.81 to 0.81, whereas the structural thermosensitivity (Ω_10_) was calculated to be 4.6, closely matching the expected experimental value of 3.89 (Table 1). Meanwhile, the biggest Grid_16_ appeared to regulate the weakest E561-R617 H-bond via a thermoring from F551 to E561, R617, F618, R619, F612, T604, F544, Y629, and back to F551 (Figure 5B–C). Because this weakest bridge between two pore turrets was energetically equivalent to approximately 1.8 basic H-bonds (1.8 kcal/mol), the theoretical T_m,th_ was estimated to be 40 °C, placing it within the 31–40 °C threshold range associated with the second heat activation (Table 1).

Therefore, this 2-APB/PBC-activated state at low temperature may serve as a pre-open activated state that facilitates the use-dependent heat activation and enhances run-up in rTRPV2 (6). Notably, with PE reinstated in the VSLD (Figure 5A), the swapped Y625-R639’ bridge near the lower gate, together with the P499-F618’ and L594-F612’ bridges near the upper gate, was restored when compared with the pre-open closed state (Figure 5D) (34). These observations are consistent with the cryo-EM structure, which exhibits a widened lower gate and a relatively narrow upper gate (47).

## DISCUSSION

The three-state model has been proposed to explain the run-up of rTRPV2 induced by either 2-APB or heat. However, the role of inactivation in these processes remains unclear. In this computational study, the highly sensitive thermoring energetic model was first used to analyze and compare several state-dependent cryo-EM structures obtained under different chemical perturbations at the same low temperature. The model then identified three key conformational states—a pre-open closed state, a pre-open inactivated state and an activated (open) state —with matched thresholds, distinct systematic thermal instabilities, and comparable thermo-sensitivities that account for 2-APB-induced run-up and mirror the fundamental features of heat-induced run-up. In addition, a pre-open activated state was identified, along with a lower temperature threshold and a reduced thermo-sensitivity to align with the measurements, providing a mechanistic explanation for the enhanced run-up induced by heat.

The results further indicate that, although the first irreversible, rate-limiting step from the closed state to the thermally unstable inactivated state is essential for run-up, the greater dynamic systematic thermal instability of the inactivated state relative to the activated state favors run-up induced by ether 2-APB or heat. Most notably, initial ligand-free inactivation was associated with an opposite pattern of allosteric gating coupling between the weakest tertiary noncovalent bridge and the intersubunit interactions near the lower gate. These findings advance our understanding of the role of the ligand-independent allosteric communication between the weakest tertiary domain-domain interface and the quaternary intersubunit interface in regulating TRPV2 activity.

### Run-up induced by 2-APB and heat shares the same pre-open closed state

**Figure 6.**
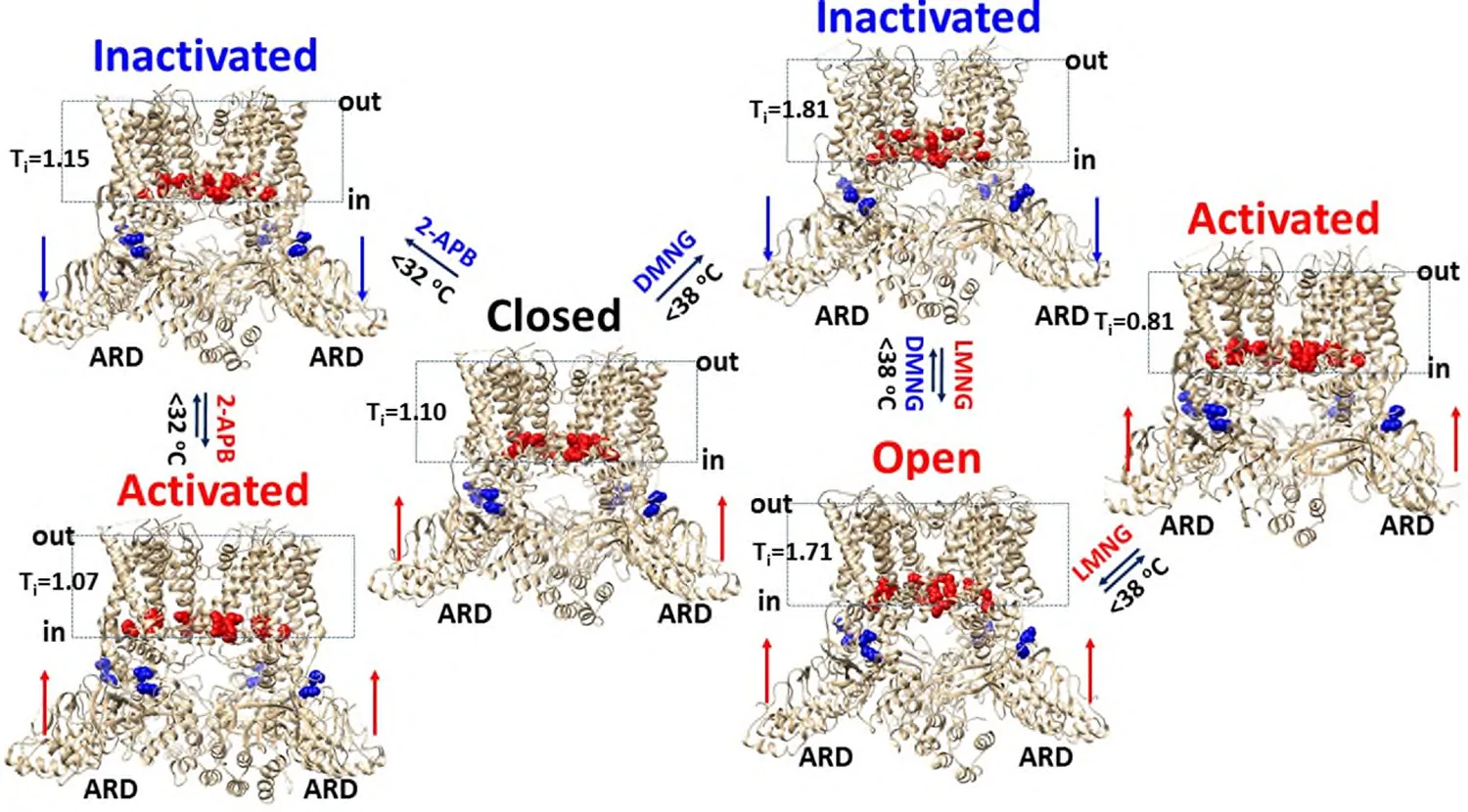
Working model of the activation and inactivation of rTRPV2 by 2-APB and mild detergents. The homo-tetrameric cryo-EM structures of rTRPV2 in the PE-bound closed state 3, the PE-free activated and inactivated states upon 2-APB perturbations at 4 °C (PDB ID, 8EKR, 7N0N and 7N0M, respectively), together with 2-APB/PBC-activated, DMNG-inactivated and LMNG-opened states (PDB ID, 9B3V, 5HI9 and 6BO4 respectively), are utilized for the run-up model. In the closed state with the ARD-up, the weakest intrasubunit K300-N354 bridges (blue) at the pre-S1/ARD interfaces are present along with swapping H521-R539’-Y525-N639’ bridges (red) near the lower gate. When the channel is activated by 2-APB or LMNG, these swapping bridges are broken. Simultaneously, disrupting the weakest K300-N354 bridges by 2-APB or DMNG inactivates the channel and links Y525 and R539’ together, along with the ARD-down. However, the higher systematic thermal instability (T_i_) of the inactivated state than the activated or open state still primes run-up.

A recent study identified a putative pre-open closed state with a matched melting threshold (T_m,th_) of 47.6–55 °C during the initial heat activation and a corresponding cold sensitivity of 126, mirroring primary heat activation above 48 °C (34). According to the heat capacity model (71), this pre-open closed state also serves as the primary starting point for heat-induced run-up (Figure 6) (6). Consistent with experimental observations, the prior heat activation before 2-APB activation is the same as the initial one for heat-induced run-up (6, 34). Thereby, the initial pre-open closed state proposed for heat-induced run-up can likewise account for the run-up induced by 2-APB (Figure 6) (34).

### Inactivation causes the first irreversible step for run-up induced by heat or 2-APB

In this study, the 2-APB-inactivated state, once identified as the intermediate closed state during run-up, also exhibited a higher T_m,th_ of 49 °C for subsequent heat activation together with a matched cold sensitivity of 127, consistent with the sharp mirrored heat activation observed after maximal 2-APB activation (Table 1) (6).

Similarly, the DMNG-inactivated state generated at low temperature exhibited a T_m,th_ of 38 °C, enabling initial cold activation at temperatures below the native heat threshold of 47.6–55 °C (6, 34). Therefore, this state can likewise function as the pre-open inactivated state to mirror the first irreversible step for heat-induced run-up (Figure 6).

### Higher dynamic systematic instability of the pre-open inactivated state primes a population shift for run-up

In this study, the inactivated state induced by 2-APB or DMNG was unexpectedly identified as an intermediate state preceding channel activation. However, its higher dynamic systematic thermal instability (T_i_), 1.15 for the former or 1.81 for the latter, once partially uncoupled to the static local stability (67), still allowed the dynamic equilibrium to shift toward the 2-APB or LMNG-activated state with the T_i_ of 1.07 or 1.71, respectively, thereby facilitating run-up (Table 1; Figure 6). Given that the LMNG-opened state at low temperature mirrors the heat-evoked open state (34), the heat-induced pre-open inactivated state is likewise expected to possess a higher T_i_ enabling heat-induced run-up.

Furthermore, human TRPV2 (hTRPV2), which is unresponsive less than 1 mM 2-APB, temperatures below 61 °C, or 30 mM HOAc at pH 5.0, can be sensitized by oxidation of M528, M607 or both following treatment with the oxidant chloramine-T (ChT) (19, 73–75). This observation suggests that hTRPV2 preferentially resides in a more stable inactivated state (26). Consistent with this interpretation, systematic stabilization of the inactivated state promotes heat-induced run-down in rTRPV1, whereas systematic stabilization of the oxidized pre-open closed state facilitates heat-induced run-up in mTRPV3 (15–17).

Finally, the Q530N mutation desensitizes the 2-APB response of rTRPV2, whereas the Q530A or F472S/L510T/Q530E mutations enhance channel sensitivity to 2-APB (75). These findings suggest that Q530N may enhance inactivation by stabilizing the weakest M468-N530 bridge, whereas Q530A and F472S/L510T/Q530E may suppress inactivation by disrupting the weakest M468-Q530 bridge (Figure 2A).

### Additional pre-open activated state promotes heat-induced run-up

Although the affinity for 2-APB is slightly increased following maximal 2-APB activation, the threshold for subsequent heat activation is markedly reduced to 31–40 °C (6). Thus, while the three-state model is sufficient to explain run-up by 2-APB, an additional stable pre-open activated state with a comparable threshold (31–40 °C) and a matched thermosensitivity (4.6) is still necessary to account for the enhancement of heat-induced run-up during the second activation phase (Table 1; Figure 6).

Supporting this model, compared with the less stable reduced pre-open closed state responsible for the initial rapid activation of mTRPV3, the more stable oxidized pre-open closed state (PDB ID: 7MIN) exhibits a lower activation threshold of 40 °C and reduced thermosensitivity (2.69), characteristics that favor heat-induced run-up (16). Similarly, the TRPV2/V1(320–393) chimeric channel displays a reduced activation threshold of 42 °C and a thermosensitivity of 3.83–8.64 (76). These findings suggest that the pre-S1 domain, which interfaces with the VSLD, TRP and ARD and involves the weakest K300-N354 bridge for rTRPV2 or the weakest Y401-R499 bridge for rTRPV1 in the closed state, plays a pivotal role in the sharp heat activation of rTRPV1 or TRPV2 above 41 °C (15, 34). On the other hand, given that HoAC can sensitize rTRPV2 response to heat, pronation of H521 at the S4–S5 linker/S5’ interface may facilitate channel activation by heat (57).

### Allosteric gating coupling between the weakest tertiary bridges and the intersubunit bridges near the lower gate

Allosteric regulation of protein activity by genetic or chemical perturbations has been extensively investigated through both experimental and theoretical studies (29–33, 58–66). In contrast, the mechanisms by which physical perturbations, such as heat or cold, regulate protein function remain much less understood. A recent study proposed that heat activation of TRPV1, TRPV3, and TRPV4 channels is mediated by allosteric gating coupling between the initially disrupted weakest tertiary bridges and the final broken highly conserved intersubunit swapping bridges located near the lower gate (26).

Because allosteric signaling transduction between the initial trigger and the distant final functional site is reversible (77), perturbation of the final swapping bridges would be expected to destabilize the initial weakest tertiary bridges as well. Consistent with this prediction, disruption of the swapping Y575-K589’ bridge near the lower gate by a phosphatidylcholine (PC) lipid in the inactivated state of oxidized hTRPV3 (PDB ID: 8GKA) is accompanied by the broken weakest E467-K545 bridge (27). Similarly, the weakest P494/Y498-Y563 bridges and the swapping Y598-R612’ bridges are both disconnected in an inactivated state of amphibian TRPV4 (26, 28, 78).

Collectively, these findings support a positive allosteric gating coupling between the weakest tertiary bridges within the transmembrane domain and the intersubunit interactions at the S4–S5 linker/S5’ interface in TRPV1, TRPV3, and TRPV4. This coupling provides a structural framework for understanding how perturbations at one site can be transmitted to distant gating elements to regulate channel activation.

In this study, together with the 2-APB-induced perturbation at the interface between the S4–S5 linker and S5’, rTRPV2 adopts ARD-up and ARD-down conformations in activated and inactivated states, respectively (Figure 6). In the 2-APB-activated state of rTRPV2, the weakest K300-N354 bridge at the pre-S1/ARD interface, which is present in the putative pre-open closed state, is retained, whereas the H521-R539’-Y525-N639’ bridges near the lower gate are disrupted (Figure 1). Upon disruption of the K300-N354 bridge in the 2-APB-inactivated state, alternative H521/Y525–2-APB-R539’ π interactions are formed (Figure 2).

Similarly, in the DMNG-inactivated state, the K300-N354 bridge is absent together with the presence of the swapping conserved Y525-R539’ π bridge near the lower gate (Figure 4). In contrast, when the Y525-R539’ π bridge is disrupted in the LMNG-induced open state, the K300-N354 bridge is re-established (34). These observations suggest that, in the absence of ligand, the K300-N354 bridge and the swapping Y525-R539’ π bridge are negatively allosterically coupled during rTRPV2 gating.

Because hTRPV2 is unresponsive to heat below 61 °C (19, 73, 74), the corresponding K299-N352 bridge may likewise be disrupted in the inactivated state of hTRPV2 (Table 3) (26). Further experiments such as salt-bridge engineering or disulfide trapping are necessary to examine this proposed allosteric gating coupling between the K300-N354 bridge and the Y525-R539’ π bridge (28).

### Merit and Limitations of the thermoring energetic model

The thermoring energetic model has been applied to estimate the static stability, the dynamic systematic thermal instability of protein in different functional states, as well as the structural thermosensitivity between two functional states. When these calculated parameters agree with the experimental measurements, the model provides a direct link between static structural information obtained from cryo-EM or X-ray crystallography and dynamic experimental observations. This capability has been demonstrated for understanding the activation and inactivation mechanisms of thermosensitive TRPV1–4 and cystic fibrosis transmembrane conductance regulator (CFTR) channels, as well as aldolases (15–17, 26–27, 34, 38–46). Consequently, much as the DNA double-helix model laid the foundation for modern molecular biology, the thermoring energetic model offers a cost-effective framework for connecting protein structure with thermal function and has the potential to become an important conceptual foundation for modern biochemistry and biophysics.

Despite these advantages, the model is currently limited by the spatial resolution of available structural data. For example, at low temperatures, water molecules can form H-bonds with charged or polar residues at the protein–water interface, thereby influencing the thermoring energetic architecture. However, these interfacial water molecules are often not resolved in most cryo-EM structures because of their limited spatial resolution, particularly under cryogenic conditions. As a result, their energetic contributions are not fully incorporated into the current model, representing an important limitation that may be addressed as structural resolution continues to improve.

## CONCLUSIONS

Protein activity can be either upregulated or downregulated by diverse physical and chemical stimuli, involving multiple functional states. Although high-resolution structural data have become increasingly available, accurately defining the functional states of structures and reconstructing their state-transition timelines for specific biological events remains a significant challenge.

In this computational study, the tertiary structural changes of TRPV2 during the transition from the inactivated state to the activated state in response to chemical perturbations were analyzed using a highly sensitive thermoring energetic model. Given the distinct state-dependent thermoring energetic structures under different stimulation conditions, the unique weakest interdomain H-bond, as revealed in the pre-open closed state, was found to allosterically couple the highly conserved domain-swapping interactions near the lower gate, thereby facilitating the ligand-free gating transition from the inactivated state to the open state.

Furthermore, differences in the channel’s dynamic systematic stability revealed two distinct use-dependent activation pathways mediated by an agonist and heat. These findings demonstrate that the thermoring energetic model provides a powerful framework for elucidating the allosteric coupling between protein stability and function, while also offering mechanistic insights into the precise modes of action of allosteric modulators and drugs.

## Supplementary Material

Supplementary Files

This is a list of supplementary files associated with this preprint. Click to download.

- Supplementarymaterial2APBinactivationinTRPV2R2.2.pdf

This article contains supplementary material (Tables S1, S2, S3 and S4).

## Figures and Tables

**Table 1.** Comparison of ligand-induced thermoring structural changes of rTRPV2 along the PE-dependent minimal gating pathway from V254 to P726. The comparative parameters are highlighted in bold.

PDB ID	7N0N	7N0M	5HI9	6BO4	9B3V
Lipid in the VSLD	Free	Free	Free	Free	PE
Lipid at the active vanilloid site	Free	Free	Free	Free	Free
Lipid environment	MSP2N2	MSP2N2	DMNG	LMNG	MSP2N2
Sampling temperature, °C	4	4	4	4	4
Ligand	2-APB	2-APB	Free	Free	2-APB+PBC
Gating state	Activated	Inactivated	Inactivated	Open	Activated
# of the biggest Grids	Grid_17_	Grid_10_	Grid_18_	Grid_14_	Grid_16_
grid size (s)	17	10	18	14	16
# of basic H-bonds (n) in stability equivalent to the weakest noncovalent bridge	1.0	1.5	2.0	1.5	1.8
Total non-covalent interactions (N)	104	104	78	55	121
Total grid sizes (S), a.a	111	120	141	94	98
Systemic thermal instability (T_i_)	**1.07**	**1.15**	**1.81**	**1.71**	**0.81**
T_i,Inact _-T_i,Act_	**0.08**		**0.10**		
Calculated T_m,th,_ °C at E=1 kcal/mol	**30**	**49**	**38**	**41**	**40**
Measured T_th_, °C	**22–48**	**49**	**22–48**	**22–48**	**31–40**
Calculated Ω_10_, mean at E=1.0 kcal/mol		**127**	**88.5**		**4.6**
Measured Q_10_		**143**	**3.89**		**3.89**
Refs for T_th_ and Q_10_	6	6	6	6, 26	6

**Table 2.** Interdomain bridges in various gating states of rTRPV2. The unshared bridges between activated and inactivated states are highlighted in bold.

PDB ID	ligand	Gating state	Interdomain bridges
7N0N (37)	2-APB	Activated	**K300-N354**, K304-C364, G306-N368, **F311-M372, Q316-Y343**, S327-R702, F330-V691, **E332-R706**, P337-W712, **P337-W715**, D349-R684, **S350-E672**, W351-R684, **E358-R459**, F362-R459, F362-F462, R369-D469, H370-S658, L381-F705, W386-K664, **R388-E685**, **R388-H683**, F394-F519, **Y447-Y675**, Y447-W676, W454-W676, R458-E670, **R459-E670, Y514-S667**
7N0M (37)	2-APB	Inactivated	**N259-N368, A303-C364**, K304-C364, G306-N368, **Q316-E376, P325-R702**, S327-R702, F330-V691, **T331-L722**, **E332-R687**, **E332-S723, W333-P726**, P337-W712, **D344-R702**, D349-R684, W351-R684, F362-R459, F362-F462, R369-D469, H370-S658, L381-F705, **K385-E685**, W386-K664, **R392-Y675**, F394-F519, Y447-W676, **Y447-W677**, W454-W676, **M468-Q530**
7T37 (37)	2-APB/CBD	Activated	**K300-N354, E332-R706**, **R388-E685**, **R459-E670**
7T38 (37)	2-APB/CBD	inactivated	**A303-C364**, **Q316-E376, E332-R687**, **W333-P726**, **R392-Y675**, **Y447-W677**
9B3V (47)	2-APB/PBC	Activated	**K300-N354**

**Table 3.** Identification of gating states of TRPV2 with or without a ligand.

Species	PDB ID	Nanodiscs or MβCD	PE/VSLD	PE/VBP	Ligand	Pre-S1/ARD interface	Gating state	Ref
Rabbit	5AN8	−	−	−	−	−	Inactivated	48
**Rat**	**5HI9**	−	−	−	−	−	**Inactivated**	49
Rat	6BO4	−	−	−	−	K300-N354	Open	50
Rat	6U84	+	−	−	−	−	Inactivated	51
Rat	6U86	+	−	−	−	K300-N354	Closed	51
Rat	6U8A	+	−	−	CBD-1	K300-N354	Activated	51
Rat	6U88	+	−	−	CBD-2	K300-N354	Activated	51
Rat	6WKN	+	−	−	PL(antagonist)	K300-N354	Inhibited	52
Mouse	7XEM	−	−	CHL	−	K295-N349	Inhibited	53
Mouse	7XEO	+	−	−	−	K295-N349	Closed	53
Mouse	7XEU	−	−	−	E2	K295-N349	Activated	53
Mouse	7XEV	−	−	2-APB	E2	−	Inactivated	53
Mouse	7YEP	+	−	2-APB	−	−	Inactivated	53
Rat	7ZJD	−	−	−	C16-1	K300-N354	Activated	54
Rat	7ZJE	−	−	−	C16-2	K300-N354	Activated	54
Rat	7ZJG	−	−	−	PBC	K300-N354	Activated	54
Rat	7ZJI	−	−	−	C16+PBC-1	K300-N354	Activated	54
Rat	7ZJH	−	−	−	C16+PBC-2	K300-N354	Activated	54
Rat	8SLX	+	−	−	1 CBD	K300-N354	Activated	55
Rat	8SLY	+	−	+	2 CBD	K300-N354	Activated	55
Rat	8FFL	+	+	−	RR	K300-N354	Activated	56
Rat	8FFM	+	+	−	RR+2-APB	K300-N354	Activated	56
Rat	8EKP	+	+	+	−	K300-N354	Closed	57
Rat	8EKQ	+	+	+	−	K300-N354	Closed	57
Rat	8EKR	+	+		−	K300-N354	Closed	57
Rat	8EKS	+	+	−	H^+^	K300-N354	Activated	57
Rat	9B3U	+	−	−	PBC	K300-N354	Activated	47

## Data Availability

All data generated or analysed during this study are included in this published article and supplementary material.

## ABBREVIATIONS

2-APB: 2-aminoethoxydiphenyl borate
ARD: Ankyrin repeat domain
CBD: cannabidiol
CFTR: cystic fibrosis transmembrane conductance regulator
CHL: cholesterol
ChT: chloramine-T
cryo-EM: cryogenic electron microscopy
CTD: C-terminal domain
DMNG: decyl maltose neopentyl glycol
E2: estradiol
LMNG: lauryl maltose neopentyl glycol
MSP2N2: membrane scaffold protein 2N2
MβCD: methyl-β-cyclodextrin
PE: phosphatidylethanolamine
PC: phosphatidylcholine
C16: phytocannabinoid Δ9-tetrahydrocannabiorcol
PL: piperlongumine
PD: pore domain
PH: pore helix
PBC: probenecid
RR: 2-aminoethoxydiphenyl borate
T_i_: systematic thermal instability
T_m,th_: melting temperature threshold
TRP: transient receptor potential
TRPV: TRP vanilloid
TRPVi (i=1, 2, 3, 4)
hTRPV1: human TRPV1
hTRPV2: human TRPV2
hTRPV3: human TRPV3
hTRPV4: human TRPV4
mTRPV3: mouse TRPV3
rTRPV1: rat TRPV1
rTRPV2: rat TRPV2
rTRPV4: rat TRPV4
VSLD: voltage-sensor-like domain
